# Genetic and Molecular Characterization of a Self-Compatible *Brassica rapa* Line Possessing a New Class II *S* Haplotype

**DOI:** 10.3390/plants10122815

**Published:** 2021-12-20

**Authors:** Bing Li, Xueli Zhang, Zhiquan Liu, Lulin Wang, Liping Song, Xiaomei Liang, Shengwei Dou, Jinxing Tu, Jinxiong Shen, Bin Yi, Jing Wen, Tingdong Fu, Cheng Dai, Changbin Gao, Aihua Wang, Chaozhi Ma

**Affiliations:** 1 National Sub-Center of Rapeseed Improvement in Wuhan, National Key Laboratory of Crop Genetic Improvement, College of Plant Science and Technology, Huazhong Agricultural University, Wuhan 430070, China; 18735424247@163.com (B.L.); 15110670184@163.com (L.W.); 15071303711@163.com (X.L.); doushengwei@webmail.hzau.edu.cn (S.D.); tujx@mail.hzau.edu.cn (J.T.); jxshen@mail.hzau.edu.cn (J.S.); yibin@mail.hzau.edu.cn (B.Y.); wenjing@mail.hzau.edu.cn (J.W.); futing@mail.hzau.edu.cn (T.F.); cdai@mail.hzau.edu.cn (C.D.); 2 Wuhan Vegetable Research Institute, Wuhan Academy of Agricultural Sciences, Wuhan 430345, China; zywweirui@sina.cn (X.Z.); lp19871120@126.com (L.S.); 3 Hunan Vegetable Research Institute, Hunan Academy of Agricultural Science, Changsha 410125, China; lzq0826@163.com

**Keywords:** *Brassica rapa*, self-incompatibility/self-compatibility, *S* haplotype, *S* locus receptor kinase (SRK), *S* locus cysteine-rich protein (SCR), amino acid variations

## Abstract

Most flowering plants have evolved a self-incompatibility (SI) system to maintain genetic diversity by preventing self-pollination. The *Brassica* species possesses sporophytic self-incompatibility (SSI), which is controlled by the pollen- and stigma-determinant factors SP11/SCR and SRK. However, the mysterious molecular mechanism of SI remains largely unknown. Here, a new class II *S* haplotype, named *BrS-325*, was identified in a pak choi line ‘325’, which was responsible for the completely self-compatible phenotype. To obtain the entire *S* locus sequences, a complete pak choi genome was gained through Nanopore sequencing and de novo assembly, which provided a good reference genome for breeding and molecular research in *B. rapa*. *S* locus comparative analysis showed that the closest relatives to *BrS-325* was *BrS-60*, and high sequence polymorphism existed in the *S* locus. Meanwhile, two duplicated *SRKs* (*BrSRK-325a* and *BrSRK-325b*) were distributed in the *BrS-325* locus with opposite transcription directions. *BrSRK-325b* and *BrSCR-325* were expressed normally at the transcriptional level. The multiple sequence alignment of SCRs and SRKs in class II *S* haplotypes showed that a number of amino acid variations were present in the contact regions (CR II and CR III) of BrSCR-325 and the hypervariable regions (HV I and HV II) of BrSRK-325s, which may influence the binding and interaction between the ligand and the receptor. Thus, these results suggested that amino acid variations in contact sites may lead to the SI destruction of a new class II *S* haplotype *BrS-325* in *B. rapa*. The complete SC phenotype of ‘325’ showed the potential for practical breeding application value in *B. rapa*.

## 1. Introduction

In flowering plants, self-incompatibility (SI) evolved to maintain genetic diversity and prevent self-pollination, in which pistils inhibit pollen germination or pollen tube growth after self-pollination [1]. In *Brassica* species, the SI system is characterized by sporophytic self-incompatibility (SSI), in which the diploid parental genotype determines the pollen grain self-incompatible phenotype, and is controlled by a single Mendelian locus (named *S* locus) [2]. The *S* locus contains multiple physically linked genes and segregates with the SI phenotype [3]. The *S* locus genes have been identified, including *S* locus Glycoprotein (*SLG*), *S* locus receptor kinase (*SRK*), and *S* locus cysteine-rich protein (*SCR*)/*S*-locus protein 11 (*SP11*). *SLG* was the first identified *S* locus gene, encoding a protein secreted in stigmatic papilla cells [4] and may play a role as an enhancer of SI in *Brassica* [5,6,7]. *SRK* was the second identified gene, and encodes a transmembrane serine/threonine receptor kinase located at the stigmatic papilla cell plasma membrane, which had been testified as being the female determinant factor [8,9,10,11]. *SP11/SCR* was the third identified gene in the *S* locus, and encodes a peptide protein located in anther tapetum cells and/or pollen grains, which had been testified as being the male determinant factor [12,13,14]. When pollen comes into contact with the pistil with the same *S* haplotype, SRK is activated and a signaling cascade is elicited within the stigmatic papilla cell to reject self-pollen [15,16]. 

Based on the nucleotide sequence similarity, *S* haplotypes were classified into two groups, class I and class II [17]. Class I *S* haplotypes are dominant over class II in pollen and the dominance relationship of *S* haplotypes is involved in *SCR/SP11* expression suppression, which is controlled by the *SCR* methylation inducer (*Smi*) [18,19,20,21,22]. However, a codominant relationship of *S* haplotypes is observed in pistils [23,24]. More than 100 *S* haplotypes have been identified in *B. rapa* [25], and about 50 *S* haplotypes have been reported in *B. oleracea* [26], while several *S* haplotypes have been identified in *B. napus*, including *BnS-1* to *BnS-7* and *BnS-1300* [27,28]. However, an interesting phenomenon has been observed, in that there are just four class II *S* haplotypes in *B. rapa,* including *BrS-40, BrS-60, BrS-29*, and *BrS-44* and three class II *S* haplotypes in *B. oleracea*, including *BoS-2b, BoS-5,* and *BoS-15* [29]. A linear dominance hierarchy among class II *S* haplotypes has been observed through reciprocal test pollinations in *B. rapa* (*BrS-44* > *BrS-60* > *BrS-40* > *BrS-29*) [19,23], and these are controlled by *SCR* methylation inducer 2 (*Smi2*) [21]. Some partial genomic regions containing *SCR* and *SRK* genes in the recessive class II *S* haplotypes have been characterized in *B. rapa* [30,31]. However, the report regarding the comparatively complete *S* locus organization of the class II *S* haplotypes was limited to the *BrS-60* haplotype [32,33], which extremely limited the understanding of the *S* locus.

In the last few decades, researchers have concentrated on identifying the SI reaction factors; however, the self-incompatible mechanism and self-compatible pathway remain unexplained. Mutant screening and research are very valuable to identify new factors involved in the SI system [34,35,36,37,38]. Self-compatible mutants are mainly attributed to the disruption of *S* locus genes, including *SCR* genes [27,39,40] and *SRK* genes [38,41,42]. On the other hand, non-*S* locus gene disruption also changes the SI phenotypes, such as M-locus protein kinase (MLPK) [36,43,44] and ARC1 (Armadillo repeat-containing protein 1(ARC1) [45,46,47,48], which are the self-incompatible factors. With the development of molecular technology and sequencing strategy, transcriptomicse and proteomics have promoted SI signal pathway research [49,50,51]. Some genes, such as exocyst complex subunit A1(*EXO70A1*), *Phospholipase D α1(PLDα1*), and *Glyoxalase1(GLO1*), were identified as self-compatible factors working downstream of ARC1 [47,52,53,54,55]. The GATA transcription factor BnA5.ZML1 participated in SI responses by suppressing the expression of SI responsive genes [56]. Thus, the SI system is a complex and mysterious biological phenomenon and much more work is needed to discover its molecular mechanism.

The screening and establishment of new self-compatible (SC) lines are critical for SI system research in *Brassica*. Some SC lines have been identified from SI populations [25,35,38,57]. Mutant identification and map-based cloning is a classical genetic method to identify new genes for phenotype mutation [58,59,60,61]. Recently, genome sequencing and transcriptome sequencing accelerated the cloning of genes greatly [62,63,64,65]. Several high-quality assembled genomes have been produced in *Brassica rapa* subspecies, including heading Chinese cabbage, yellow sarson, and no-heading pak choi [32,33,66], and these high-quality assembled genomes could accelerate the progress in *Brassica* genomic variation research. In this study, a completely self-compatible line with a single *S* locus mutation was identified, which had a new class II *S* haplotype, named *BrS-325*. Complete genome assembly was gained by means of Nanopore sequencing strategy and the *S*-intergenic region was determined. The *S* locus genes were identified, and *BrSCR-325* and *BrSRK-325b* had normal expression levels. Previous reports investigated leaf development, flowering, and LTR-RT expansion based on the assembled genomes in *B. rapa* [32,33,66]; meanwhile, we concentrated on performing comparative analysis of the *S* locus of two class II *S* haplotypes, *BrS-60* and *BrS-325,* which are each other’s closest relatives. The *S* locus of *BrS-60* and *BrS-325* showed high sequence polymorphism with few genes’ distribution. Meanwhile, multiple sequence alignment of class II SCRs and SRKs was carried out to reveal amino acid variations in the contact regions (CR II and CR III) of BrSCR-325 and the hypervariable regions (HV I and HV II) of BrSRK-325s, which may influence the interaction between the ligand and the receptor. Here, a complete pak choi genome was obtained and many amino acid variations were identified in the contact regions of BrSCR-325 and the hypervariable regions of BrSRK-325s, which may lead to self-incompatibility destruction.

## 2. Results

### 2.1. Determination of a New Class II S Haplotype in an SC B. rapa

**Figure 1:**
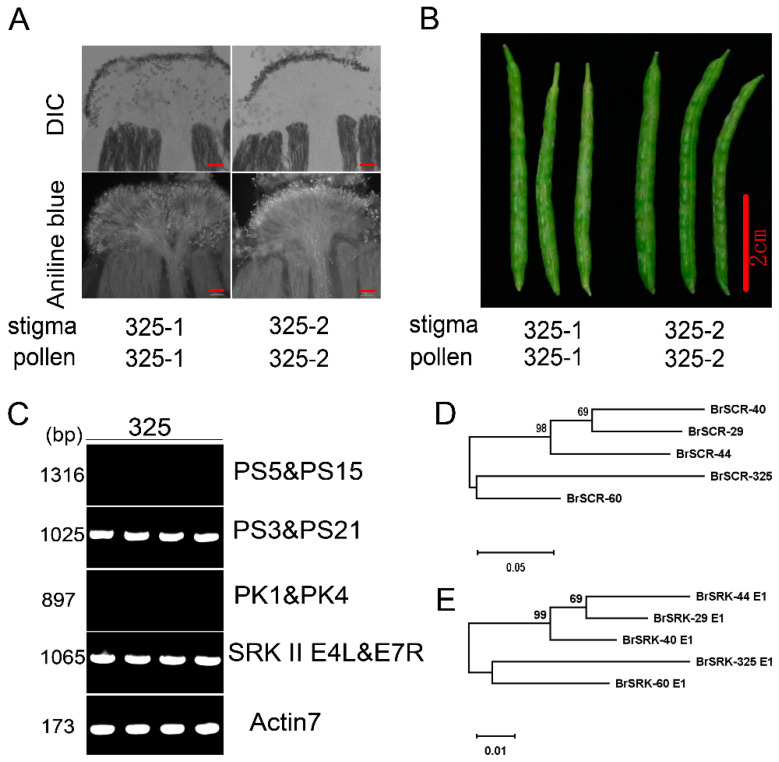
Self-compatibility phenotype identification and *S* haplotype determination of the inbred line ‘325’. (**A**) self-pollination of inbred line ‘325’ by observing with aniline blue staining, scale bar = 100 μm; (**B**) self-pollination of inbred line ‘325’ by observing silique development, scale bar = 2 cm; (**C**) *S* haplotype identification with class I and class II universal primer pairs, PS5 and PS15 and PK1 and PK4 were used for class I *S* haplotypes identification, PS3 and PS21 and SRK IIE4L and E7R were used for class II *S* haplotypes identification, with *Actin7* as the control. (**D**,**E**) phylogenetic analysis of class II *S* locus genes *SCR* and *SRK* E1. An unrooted phylogenetic tree was constructed using the neighbor-joining method. Bootstrap values from 1000 replicates are shown.

In our breeding process, we found that the inbred line ‘325’ was completely self-compatible, the self-pollination of which involved normal pollen germination and produced normal siliques, harboring full seeds (Figure 1A,B). To explore the mechanism of self-compatibility in ‘325’, we determined the *S* haplotype by cloning and analyzing *S* locus genes. The line ‘325’ could be amplified by universal primer pairs of class II, but not class I, indicating that a class II *S* haplotype existed (Figure 1C). Then, based on the sequences of class II *SRK* and *SCR* genes, partial sequences of *SCR* and *SRK* E1 (the first exon of *SRK*) were obtained by the means of PCR and sequenced (Figures S1 and S2). Nucleotide sequence comparative analysis revealed that both the *SCR* and *SRK* E1 sequences in ‘325’ were different from the reported class II *S* locus genes and showed the highest sequence similarity with that of *BrS-60* (79% sequence similarity of *SCR* and 91% sequence similarity of *SRK* E1) (Figures S1 and S2). Phylogenetic analysis results also showed that both the *SCR* and *SRK* E1 genes in ‘325’ were the closest relatives to those of *BrS-60* (Figure 1D,E). Thus, a new class II *S* haplotype, named *BrS-325*, was determined.

### 2.2. Genetic Analysis of SC Trait in ‘325’

**Figure 2:**
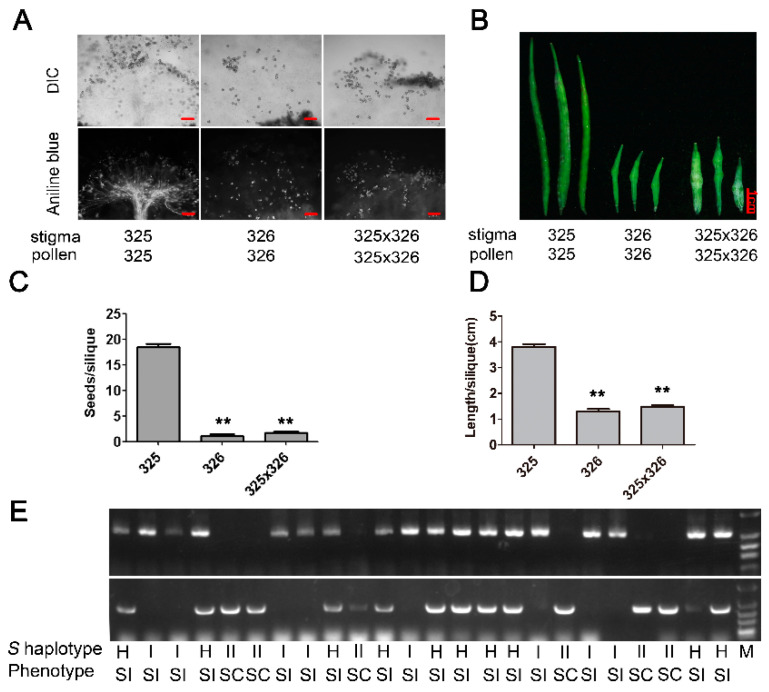
Self-pollination phenotype analysis and *S* haplotype identification of the segregation population derived from SC ‘325’ and SI ‘326’. (**A**) Aniline blue staining observing pollen germination and pollen tube growth of the self-pollinated SC line ‘325’, SI line ‘326’ and hybrid F_1_ coming from the SC parent ‘325’crossed with the SI parent ‘326’. Scale bar = 100 μm; (**B**) silique development of self-pollinated the SC line ‘325’, SI line ‘326’ and hybrid F_1_. Scale bar = 1 cm; (**C**) quantification of silique length of the SC line ‘325’, the SI line ‘326’ and the hybrid (‘326’ × ’325’) after self-pollination; (**D**) seeds per silique of the SC line ‘325’, the SI line ‘326’ and the hybrid (‘325’ × ’326’) after self-pollination, *n* = 10 siliques. Error bars indicate SE. The significance was analyzed by Student’s *t*-test (** *p* < 0.01); (**E**) *S* genotypes identification and SI phenotypes analysis in partial representative plants of the F_2_ population with class I (PK1and PK4) and class II (SRKII-E4Land &SRKII-E7R) *S* haplotype specific primer pairs. SC: self-compatible, SI: self-incompatible. H: class I and class II *S* haplotypes, I: class I *S* haplotype, II: class II *S* haplotype.

To reveal the genetic basis of the SC trait in ‘325’, reciprocal cross F_1_ hybrids were created from the SI parent ‘326’ (*BrS-12*) and SC parent ‘325’ (*BrS-325*), and pollination behavior displayed self-incompatibility (Table 1). The aniline blue staining results showed that a large amount of pollen grains germinated and passed through the stigma in the SC mutant. However, few pollen grains germinated and passed through the stigma in the SI parent ‘326’and F_1_ hybrids (Figure 2A). Siliques in SC ‘325’ were dramatically longer than that of the SI parents and F_1_ hybrids (1.29 and 1.48 cm per silique) (Figure 2B,C). Average seed-set of SI parents and F_1_ hybrids were 1.14 and 1.76 seeds per silique, respectively, which was dramatically less than the SC mutant (18.47 seeds per silique) (Figure 2B,D). Based on the reciprocal cross F_1_ hybrid phenotypes, this meant that ‘325’ had a recessive nucleus mutation. In the F_2_ population, originating from the self-pollination of the F_1_ hybrid, pollination phenotypes were segregated into SI:SC = 70:22 (3:1, χ^2^ = 0.094, *p* < 0.05), indicating that the SC trait in ‘325’ was controlled by a single genetic locus. *S* haplotypes in the F_2_ population were segregated into *S-12S-12*: *S-12S-325*: *S-325S-325* =20:50:22 (1:2:1, χ^2^ = 0.73, *p* < 0.05), and all the pollination phenotypes were consistent with the genotypes (Figure 2E and Table 1). All the results show that *BrS-325* was responsible for the SC in ‘325’. 

### 2.3. Nanopore Sequencing and De Novo Assembly the Genome of SC Line ‘325’

**Figure 3:**
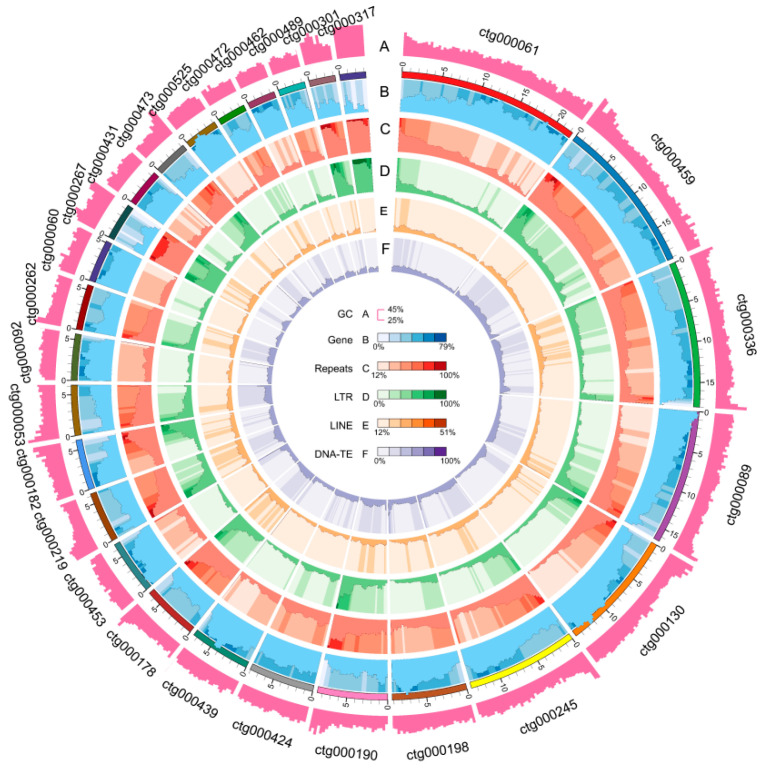
Nanopore sequencing and de novo genome assembly of the SC line’325’. Circular representation of anchored contigs of the SC line ‘325’. (**A**) GC content distribution.; (**B**) gene distribution; (**C**) repeat sequences distribution; (**D**) long terminal repeat retrotransposon (LTR) distribution; (**E**) long interspersed nuclear elements (LINE); (**F**) DNA-transposon (DNA-TE).

To obtain complete genomic information for *BrS-325* and discover the underlying mechanism of SC in ‘325’, we carried out Nanopore sequencing and next de novo assembly to obtain the pak choi genome [67]. Here, we produced a 376.70 Mb genome assembly, containing 314 contigs with an N50 size of 4.54 Mb, which was longer than previous *B. rapa* genome assemblies (Table 2, Tables S1 and S2) [66]. To determine the accuracy and completeness of the genome assembly, the second-generation reads and third-generation reads were mapped against the assembled genome. The results showed that the mapping rate reached 99.39% and 99.99%, and the coverage reached 97.79% and 99.98% (Table S3). Next, Benchmarking Universal Single-Copy Orthologs (BUSCOs) were assessed. The results showed that 97.50% were complete BUSCOs, and only 1.74% were missing BUSCOs. Respectively, 84.65% and 12.85% of the complete BUSCOs accounted for the single- and multi-copy genes (Table S4). These results suggest that we acquired a complete pak choi genome assembly (Figure 3). 

We then performed gene prediction in our assembled genome. Approximately 52.65% of the genome sequences were annotated as repetitive sequences, including DNA-TE (15.60%), retrotransposons (42.41%), and unclassified elements (3.41%). The annotated retrotransposons mainly consisted of the long terminal repeat retrotransposon (LTR-TE), which covered 120 Mb and occupied 31.96% of the assembled genome (Table S5). In total, 43,855 genes of the coding protein were identified, which had an average size of 2650 bp and consisted of an average of five exons (Table S6). About 42,500 genes could be annotated by utilizing the foreign protein database, which could annotate 96.91% of the predicted genes (Table S7). Meanwhile, a total of 1431 tRNA, 1623 rRNA, 241 microRNA (miRNA), and 1397 small nuclear RNA (snRNA) genes were identified (Table S8). To evaluate the accuracy and completeness of the gene annotation, we carried out BUSCO assessment. The results showed that approximately 97.4% of the embryophyte genes could be detected in our pak choi genome (Table S9). These results indicate that we gained a complete and functionally annotated pak choi assembled genome. 

### 2.4. S Locus Organization of BrS-325 

**Figure 4:**
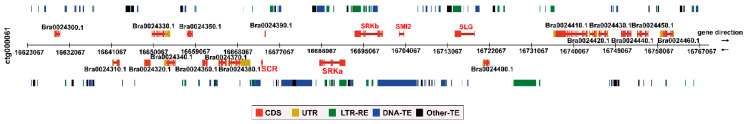
*S* locus organization of *BrS-325.* The 145 kb sequence of ctg00061 covered the whole *BrS-325* locus. The red and orange boxes indicate the coding sequence (CDS) and un-translated region (UTR), respectively. The green and blue boxes indicate the long terminal repeat retrotransposon (LTR-TE) and DNA-transposon (DNA-TE) and the black box indicate the other-TE (unclassified elements).

Based on the cloned sequences of *SCR* and *SRK* from ‘325’, homologous genes were blasted in our assembled genome. Finally, we obtained a 145 kb sequence from contig, named ctg00061, which covered the whole *BrS-325* locus and boundary regions (Figure 4). In ctg000061, the *SCR* homologous gene *BrSCR-325* was found to be located 16,673,067 to 16,673,465 bp of the assembled genome, consisting of two exons and one intron. Meanwhile, we blasted the *SLG* homologous sequence of *BrSLG-60* [32] in ctg000061, and *BrSLG-325* was identified 41.173 kb downstream of *BrSCR-325*, which contained two exons and one intron. Interestingly, two *SRK* genes, named *BrSRK-325a* and *BrSRK-325b*, were found to be located in the intergenic region between *BrSCR-325* and *BrSLG-325*. Both *BrSRK-325a* and *BrSRK-325b* consisted of seven exons and six introns, coding 855 AA and 856 AA, respectively. There were just 61 different amino acids in BrSRK-325a and BrSRK-325b (Figure S4B). They showed opposite transcriptional directions and shared a 280 bp common promoter in a 1946 bp intergenic region (Figure S3). Based on the precursor sequences of *BrS60-Smi* and *BrS60-Smi2*, *BrS60-SMI* and *BrS60-SMI2* [21], respectively, we blasted the homologous sequence in ctg000061, and only *BrS325-SMI2* was identified. In addition, many repeat sequences and genes were also annotated in the upstream and downstream regions of the *S* locus (Figure 4). In a word, we successfully identified the SI-related genes in *BrS-325*, including one *BrSCR-325*, one *BrSLG-325*, and two *SRK* genes (*BrSRK-325a* and *BrSRK-325b*) (Figure 4 and Table S10). 

### 2.5. Comparative Analysis of the S locus of Two Class II S Haplotypes

**Figure 5:**
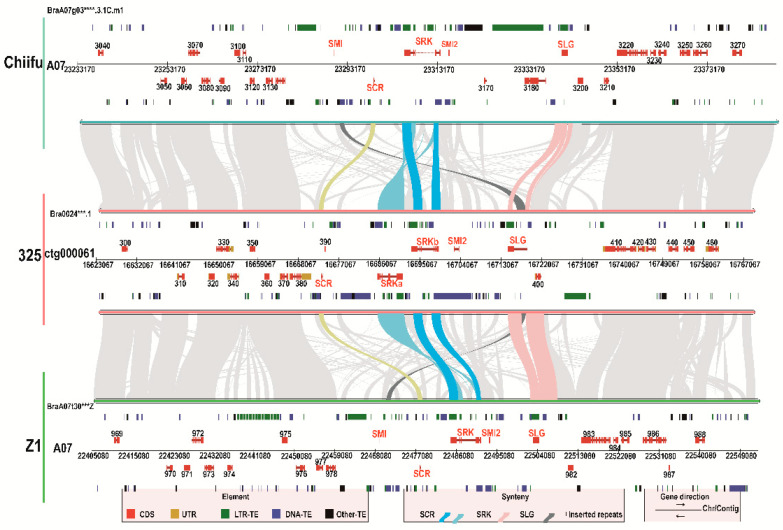
Comparative analysis of the genomic organization of the *S* locus between two class II *S* haplotypes, *BrS-60* and *BrS-325.* Red and orange boxes represent CDS and UTR, respectively, and repetitive sequences (LTR-TE, DNA-TE and other-TE) are represented by green, blue, and black boxes. The SI genes *SCR*, *SRK* and *SLG* are shown by gene names, with *SMI1* and *SMI2* representing the *S* haplotype-linked small RNAs involved in dominance interactions.

As the SI genes in *BrS-325* showed the highest sequence similarity with those of *BrS-60* (Figures S1 and S2), we carried out *S* locus comparative analysis between them. By means of a homologous sequence alignment, two sequences covering the whole *S* locus of *BrS-60* were obtained; one had a length of 147 kb from *B. rapa* cultivars ‘Z1’ [33] and the other had a length of 155 kb from *B. rapa* cultivars ‘Chiifu’ [32] (Table 3), in which contrast phenotypes were not attributed to the *S* locus variation [68]. Previous reports suggested that high sequences polymorphism existed in the *S* locus region, while sequences outside the boundary were highly conservative [69]. Through sequence alignment and collinearity analysis, the *S* locus was determined. The *S* locus of *BrS-325* covered 67.777 kb, and that of *BrS-60* covered 73.764 kb and 54.448 kb in ‘Chiifu’ and ‘Z1’, respectively (Table 3). In the *S* locus regions, except for *BrSRK-325a*, the distribution of SI-related genes was completely conserved, with *SCR* lying upstream of *SRK*, and *SLG* lying downstream of *SRK* (Figure 5). Additionally, all the SI-related genes showed conserved transcription directions (Figure 5). By comparing the intergenic region between *SCR* and *SLG*, we found that the spacing distance reached 41.173 kb, containing two duplicated *SRK* genes in the *BrS-325* locus, while the distance reached 41.192 and 24.828 kb in the *BrS-60* locus of ‘Chiifu’ and ‘Z1’, respectively. Referring to the *BrSRK-325b* gene location in the *BrS-325* locus, the intergenic region of *SCR* and *SRK* reached 19.706 kb in the *BrS-325* locus, while an identical spacing distance was observed in the *BrS-60* locus of ‘Chiifu’ and ‘Z1’. There was a big difference in the intergenic region of *SRK* and *SLG*. The spacing distance reached 15.384 kb in the *BrS-325* locus, while the distance reached 26.943 and 11.533 kb in the *BrS-60* locus of ‘Chiifu’ and ‘Z1’, respectively (Table 3). LTR-TE, DNA-TE and other-TEs were widely distributed in the intergenic regions of *S* locus (Figure 5). Meanwhile, a DNA-TE inserted into the intron of *BrSLG-325* was identified. Taken together, similar *S*-intergenic regions with high polymorphism in the *S* locus and which were relatively conservative in boundary regions existed in different *S* haplotypes (Figure 5). All of these results further indicate that *BrS-325* is a new class II *S* haplotype.

### 2.6. Expression Analysis of SRK and SCR

**Figure 6:**
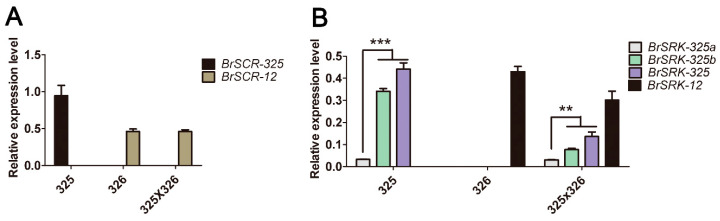
Expression analysis of the SI genes. (**A**) relative expression level of *SCR* genes in the anther. *BrSCR-325* expression was detected in ‘325’ and the F_1_ hybrid, while *BrSCR-12* expression was detected in ‘326’ and the F_1_ hybrid; (**B**) relative expression level of *SRK* genes in the stigma. *BrSRK-325a* and *BrSRK-325b* expressions were detected in ‘325’ and the F_1_ hybrid, while *BrSRK-12* expression was detected in ‘326’ and the F_1_ hybrid. *BrSRK-325* indicated that *BrSRK-325a* expression and *BrSRK-325b* expression were simultaneously detected in line ‘325’ and the F_1_ hybrid. Error bars represent standard errors (SE) of the mean of triplicate samples. The significance was analyzed by Student’s *t*-test (** *p* < 0.01; *** *p* < 0.001). The relative expression was corrected using the reference gene *Actin 7*.

Normal expression of *SRK* and *SCR* is a critical first step for the interaction between the receptor and the ligand, which could be supported by *SRK* and *SCR* expression suppression [27,39,40,70,71]. To detect whether the SC phenotype of ‘325’ was caused by a change in the expression of SI genes, RT-qPCR was performed in the SC line ‘325’, the SI line ‘326’ (*BrS-12* haplotype), and their F_1_ hybrid. *BrSCR-325* showed a higher expression level in the anther of the SC line ‘325’ than that of *BrSCR-12* in the SI line ‘326’, while in the F_1_ hybrid, the expression of *BrSCR-325* was hardly detected (Figure 6A). The silence of *BrSCR-325* in the F_1_ hybrid may be attributed to the suppression effect of the dominant class I *BrS-12* haplotype in the SI line’326’ [72]. In the stigma side of ‘325’and F_1_ hybrid, the expression of *BrSRK-325a* was hardly detected, but *BrSRK-325b* was expressed normally (Figure 6B). All the results indicate that, at the transcriptional level, the SI genes *BrSCR-325* and *BrSRK-325b* are expressed normally in SC line ‘325’. 

### 2.7. Amino Acid Sequence Alignment and Variation Analysis of Class II SRK and SCR

**Figure 7:**
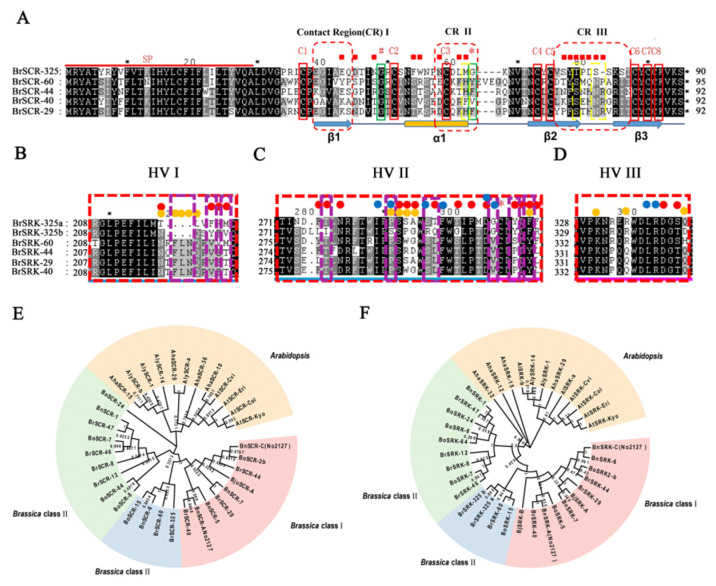
Multiple amino acid sequence alignment and Evolutionary analysis of the *S* locus gene *SRKs* and *SCRs*. (**A**) sequence alignment of class II SCRs. Above the alignment, the red line marks the signal peptide (SP). The conservative cysteine acid is shown by a red label C * and a red solid box. The red dotted boxes indicate the contact regions of class II SCRs. Amino acids in contact with eSRK are shown by red square boxes. Variable amino acids ofBrSCR-325 compared with other class II SCRs are shown by yellow dotted boxes. Arrows and cylinders indicate the ß strands and α helixes of class II SCRs, respectively; (**B**–**D**) the hypervariable regions (HV I, HV II, HV III) sequence alignment of class II SRKs. Above the alignment, amino acids in contact with the cognate SCR are indicated by red circles, based on the same sites in contact with amino acids of BrSRK-8/BrSCR-8 and BrSRK-9/BrSCR-9, and yellow circles, based on the contact amino acids of class II SRK. The blue circles indicate the residues involved in SRK homo-dimerization. The variable amino acids of BrSRK-325 compared with other class II SRK are indicated by purple dotted boxes; (**E**,**F**) An unrooted phylogenetic tree of different *S* haplotypes SCRs and SRKs in *B. rapa*, *B. oleracea*, and *B. nupus*. A phylogenetic tree was constructed using the neighbor-joining method. Bootstrap values from 1000 replicates are shown. The pink circular arc indicated the class I *S* haplotypes of *Brassica*. The light blue circular arc indicated the class II *S* haplotypes, which were relatively the closest to *BrS-325* in *Brassica*. The light green circular arc indicated the class II *S* haplotypes of *Brassica.* The orange yellow circular arc indicated the *S* haplotypes in *Arabidopsis*.

There are a limited number of class II *S* haplotypes, including *BrS-44, BrS-60, BrS-40* and *BrS-29* in *B. rapa* and *BoS-2b, BoS-5, BoS-15* in *B. oleracea* (Table S10) [73]. To understand whether amino acid sequence variations contribute to the phenotype change, we carried out the multiple sequence alignment of the class II SCR and SRK in *B. rapa.* The amino acid sequence identities among SCRs were 57% to 73% (Table S11), and among the class II SRKs, they were very conservative, from 86.67% to 94.62% (Table S12). Evolutionary analysis results showed that the phylogenetic tree was divided into two branches, *Arabidopsis* species and *Brassica* species. It meant that a closer relationship was identified in different species within the same genus. Furthermore, the branches could be divided into two groups, class I and class II, based on the sequences similarity of *Brasssica* species. The interspecific pairs were each other’s closest relatives with higher sequence similarity, and *BrS-325* was the relative closest to *BrS-60* (Figure 7E,F).

As we know, the hypervariable region of SRK and contact regions were necessary for the interaction between SCRs and SRKs with the same *S* haplotypes [73,74,75]. Based on class II eSRKs and SCRs structure models [75], we compared the contact regions (CR I, CR II, and CR III) of class II SCRs and the hypervariable regions (HV I, HV II, and HV III) of class II SRKs (Figure 7 and Figure S4). Sequence alignment showed that some variable amino acids existed in CR II and CR III of BrSCR-325, in which the amino acid sites were identical in other known class II SCRs, including amino acid alteration (e.g., Met62, Thr75, Ser78 in *BrS-325*) and amino acid deletion (e.g., Pro83 in *BrS-60* and Arg80 in *BrS-44*). 

Referring to the BrSRK-8/BrSRK-9 structure [74,75] and hypervariable regions of class II SRKs [75], we carried out amino acid sequence alignment of class II SRKs (Figure S4). The results showed that the twelve conserved cysteine residues were intact, and several amino acid variations presented in the *S* domain of BrSRK-325 (Figure S4). The amino acid variation analysis of hypervariable regions showed that HV I and HV II of BrSRK-325 had very high polymorphism, while the HV III variations had very low polymorphism (Figure 7B–D and Figure S4). It is worth noticing that three continuous amino acid deletions (e.g., Phe219, Leu220, Asn221) and two amino acid alterations (e.g., Phe220 and Met222 in BrSRK-325a and Tyr220 and Val222 in BrSRK-325b) were observed in the HV I, (Figure 7B and Figure S4B). Many variation residues were distributed in the HV II, including Ile277, Ser286, Arg291, Gln292, Gly300, Tyr303 and Phe305 in BrSRK-325b and Thr291, Gly299, Tyr302, and Phe304 in BrSRK-325a (Figure 7C and Figure S4B), which may be involved in the interaction of BrSCR-325 and BrSRK-325 and BrSRK-325 homo-dimerization. These observations indicate that these amino acid variations may influence the binding of BrSRK-325 and BrSCR-325. 

## 3. Discussion

The self-incompatible system is one of the best-known physiological outbreeding devices, which can discriminate self-/non-self pollen to maintain genetic diversity and prevent self-inflicted recessions [1]. The SI phenomenon is very significant, not only for basic research, but also for crop breeding. In the past several decades, research has mainly focused on collecting materials, identifying *S* haplotypes, self-incompatible determinant factors, and self-incompatible signal cascade factors [10,11,36,53,54,55,76,77,78]. However, the mysterious molecular mechanism of SI still remains largely unknown. In *Brassica* species, many *S* haplotypes have been identified, including more than100 *S* haplotypes in *B. rapa* [25], about 50 *S* haplotypes in *B. oleracea* [26] and several *S* haplotypes in *B. napus* [27,28]. However, there are just four class II *S* haplotypes in *B. rapa* (*BrS-44*, *BrS-60*, *BrS-40* and *BrS-29*) and three class II *S* haplotypes in *B. oleracea* (*BoS-2b*, *BoS-5,* and *BoS-15*) [29]. In this study, a new class II *S* haplotype, named *BrS-325*, was identified in a pak choi line‘325’ (Figure 1). Through Nanopore sequencing and de novo assembly strategy, we obtained the complete pak choi genome of‘325’ (Figure 3 and Table 2). The entire *S* locus of *BrS-325* was extracted from the assembled genome, and functional annotation was performed (Figure 4 and Table S10). Sequence alignment of all the class II *S* haplotypes showed that *BrS-325* had high sequence similarity with other class II *S* haplotypes. The phylogenetic tree showed that the *S* haplotypes of *Arabidopsis* species and *Brassica* species could be divided into independent branches, based on the sequence polymorphism of SCR and SRK, and *BrS-60* was the closest relative to *BrS-325* (Figure 7 and Figure S4). As seen in a previous report [31], the distribution and transcription directions of SI related genes were completely conserved in class II *S* haplotypes (Figure 5). Previous reports indicated that the genetic effects of the class II *S* haplotypes are masked, when making heterozygotes with any class I *S* haplotype [38]. In our study, *BrSCR-325* showed a high level of expression in the anther of the SC line ‘325’, while the expression of *BrSCR-325* was hardly detected in the F_1_ hybrids (created from the SI line ‘326’and the SC line ‘325’, which possess class I *BrS-12* and class II *BrS-325*, respectively) (Figure 6A). The silence of *BrSCR-325* in the F_1_ hybrids may be attributed to the suppression effect of the dominant class I *S* haplotype *BrS-12* in the SI line ‘326’ [20]. All the evidence suggests that *BrS-325* is a new class II *S* haplotype.

The rapid development of genomics technology will facilitate the exploration of the mechanisms of important trait formation in crops. In this study, the inbreed line ‘325’ was a pak choi SC mutant and possessed a new class II *S* haplotype *BrS-325* (Figure 1). Segregation analysis showed that the SC phenotype in ‘325’ was related to *BrS-325* (Figure 2 and Table 1). To obtain the *S* locus information and discover the mechanism of SC in ‘325’, a complete pak choi assembled genome was acquired by carrying out nanopore sequencing and a de novo assembly strategy (Figure 3 and Tables S1–S9). There are only a few available genome assemblies for the *B. rapa* cultivars, including the heading Chinese cabbage type, the yellow sarson oilseed type, and the no-heading pak choi, which concentrated on documenting the morphotypes’ phenotypic variations [32,33,66,79]. Some accessions were re-sequenced to investigate the domestication history in *B. rapa* [80]. Compared with the existed genome assemblies, our pak choi genome assembly had a longer N50 contig and higher coverage (Table 2, Tables S2 and S3). It provides a valuable resource for comparative genomics research and the genetic improvement of the vegetable and crop in *Brassica* [81,82,83]. According to the results, the candidate genes for leaf morphology and flowering were identified by comparing gene structure and expression in three morphotypes [66]. This is a good strategy to reveal the SI phenotype change by comparing with *S* locus variation. *BrS-325* was the closest relative to *BrS-60*, and the available genome provided the chance to compare these two class II *S* haplotypes. Based on the *SRK* and *SCR* sequences and location in the genome, we determined the *S* locus and boundary regions of *BrS-325* and *BrS-60* (Figure 4 and Figure 5). Comparative analysis showed that high collinearity emerged in the outer boundary region of *S* locus, while high sequence polymorphism existed in the *S* locus (Figure 5), which was similar to the results in *Arabidopsis thaliana* [69].

**Figure 8:**
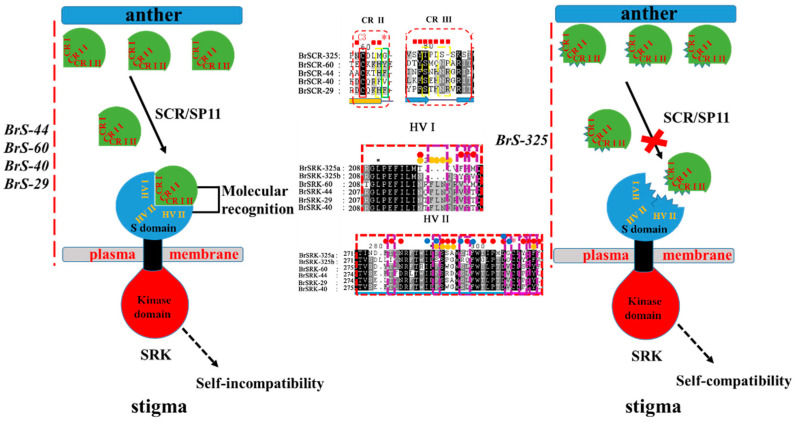
Specific recognition models of class II SRKs and SCRs. In the left upper panel, cognate SRK and SCR can specifically recognize each other in class II *S* haplotypes (*BrS-44*, *BrS-60*, *BrS-40*, and *BrS-29*), and an SI signal cascade is activated to reject self-pollination pollen. In the right upper panel, the specific recognition was destroyed for some amino acid variations, which exist in the HV I and HV II of BrSRK-325 and CR II and CR III of BrSCR-325, and SC is investigated. The red dotted box indicates the contact region of class II SCRs. Amino acids in contact with SCR and eSRK are indicated by red square boxes. Variable amino acids of BrSCR-325 compared with other class II SCR are shown by yellow dotted boxes. Above the hypervariable region sequence alignment, contact amino acids against the cognate SCR were shown red circles, based on the same sites’ contact amino acids of BrSRK-8/BrSCR-8 and BrSRK-9/BrSCR-9, and yellow circles, based on the contact amino acids of class II SRK. The blue circles indicate the residues involved in SRK homo-dimerization. The variable amino acids of BrSRK-325 compared with other class II SRK are shown by purple dotted boxes.

Several SC mutation materials were collected, and the results showed that changing the *S* locus genes, *SRK*, and/or *SCR*, leads to SI phenotype transition [27,38,39,40,42]. One *SCR* gene (*BrSCR-325*) and two duplicated *SRK* genes (*BrSRK-325a* and *BrSRK-325b*) exist in the *BrS-325* locus; however, expression analysis results showed that the expression of *BrSCR-325* and *BrSRK-325b* are normal, which may not be responsible for the SC phenotype (Figure 6). Recently, studies with a SCR and SRK structural basis have helped us to understand the specific recognition response in *Brassica* [74,75]. The specific recognition of SCR and SRK is mediated through three hypervariable regions (HV I, HV II, HV III) of eSRK and contact regions (CR I, CR II, CR III) of SCR, which predominantly contribute to ΔG for their corresponding eSRK. The specific binding induces eSRK homo-dimerization, forming a 2:2 eSRK:SCR hetero-tetramer [74,75]. Referring to the known SRK and SCR structure [74,75], we aligned the class II SRK and SCR amino acid sequences, and especially focused on the hypervariable regions of SRKs and contact regions of SCRs. The results showed that a number of amino acid variations presented in the HV I and HV II of BrSRK-325 compared to other class II SRKs (Figure 7B–D and Figure S4), which may influence the interaction between BrSRK-325 and BrSCR-325. Sequence alignment of the class II SCRs showed that some amino acid variations existed in CR II and CR III of BrSCR-325, and these mutations may influence the interaction between BrSCR-325 and BrSRK-325 (Figure 7). Thus, we speculated that amino acid variations in HV I and HV II of BrSRK-325 and CR II and CR III of BrSCR-325 may destroy the interaction between the receptor and the ligand, leading to the SI phenotype change in *B. rapa* (Figure 8). The model showed that specific recognition would occur in the contact regions of SCRs and hypervariable regions of SRKs, and lead to self-incompatibility in known class II *S* haplotypes, while the amino acid variations in contact regions and hypervariable regions may destroy the specific recognition between BrSCR-325 and BrSRK-325 in *BrS-325*. 

In the *Brassica* vegetable crop breeding process, SI is one of the major obstructions for the development of inbred lines and the propagation of parent plants. SC inbred lines are very important for the seed production of male sterile hybrid, which can greatly reduce the cost. The manipulation of *S* locus genes is one of the most widely recognized ways so far to convert SI into SC in *Brassica*. In this study, we identified a new class II *S* haplotype *BrS-325*, which had high sequence similarity with other class II *S* haplotypes, and was responsible for the complete SC of ‘325’. We concluded that *BrS-325* showed the potential for practical breeding application value in *B. rapa*.

## 4. Materials and Methods

### 4.1. Plant Materials and Growth Conditions

The self-incompatible line ‘326’, which contains a class I *S* haplotype *BrS-12* and the self-compatible line ‘325’, was provided by the Vegetable Research Institute of the Wuhan Academy of Agricultural Sciences. The self-incompatible line ‘326’ was crossed with the self-compatible line ‘325’ to produce the segregation population. All the materials were grown in a greenhouse at Huazhong Agricultural University under a light intensity of 100 μmolm^−2^ s^−1^ with a 16/8 h light/dark photoperiod at 22 °C.

### 4.2. S Haplotype Determination and Genetic Analysis

Genomic DNA from all individuals was extracted from young leaves as described by Murray and Thompson [81]. The *S* haplotypes were classified by cloning with the universal primers PS5and PS15 [82] and PK1and PK4 [83] to identify class I *S* haplotypes and PS3 and PS21 [82] and SRK II E4L and SRK II E7R to identify class II *S* haplotypes. The polymerase chain reaction (PCR) was performed in a 10 μL reaction system, including 1 μL of DNA, 0.5 μL of each primer at 10 μM, 5 μL of 2× Taq Reaction Buffer (containing Mg^2+^, dNTPs, and DNA polymerase) (Vazyme, Nanjing, Jiangsu Province, China), and 3 μL of distilled water. The amplification program involved one cycle of 95 °C for 5 min, followed by 35 cycles of 95 °C for 30 s, 56 °C for 30 s, and 72 °C for 1 min. PCR products were electrophoretically-fractionated in a 1% agarose gel, stained with ethidium bromide, and then viewed under a UV illuminator. The SCR sequence was determined using the II SCR-1Land II SCR-1R primer pair, and the partial sequence (included first exon and four to seven exons) of SRK was determined using SRK II E1L and E1R and SRK II E4L&E7R in the self-compatible line “325”. PCR products were sequenced to determine the *S* haplotype. To explain the mutation locus of the self-compatible inbred line ‘325’, the F_1_ hybrid was created by crossing the SI parent ‘326’ (*BrS-12*) and the SC parent ‘325’, and self-pollinated to produce the F_2_ segregation population. *S* haplotypes were determined with the specific primer pairs: PK1and PK4 for detecting class I *S* haplotypes and SRK II E4L and SRK II E7R for detecting class II *S* haplotypes. PCR was performed under the same conditions mentioned above. Primer information for detecting *S* haplotypes is listed in Table S14.

### 4.3. Nanopore Sequencing and Genome Assembly

#### 4.3.1. Genomic DNA Extraction, Nanopore Sequencing, and Genome Assembly

Genomic DNA was extracted from the first young leaves of the self-compatible inbred line ‘325’, according to the procedure described by Belser et al [33]. Genomic DNA quality was evaluated by detecting the purity, concentration, and integrity of genomic DNA, and a sequencing library was created according to the procedure described by Belser et al. [33]. Meanwhile, the library quality was evaluated by detecting the accurate quantification using Qubit 3.0 and the size of the library using Agilent 2100. Finally, the library was sequenced using Nanopore MinION according to the amount of target offline data [84]. After gaining sequencing data, we carried out the genome assembly using the next de novo assembly by NextDenovo tool [84,85,86,87], including, error correction, pruning, and assembly. Each step contains the following stages of data processing: (1) splitting the sequencing data according to the set number of files and converting them into bit2 format; (2) using minimap2 [88] for mutual comparison to find overlapping regions between reads and to remove redundant overlap regions; (3) calibrating the reads according to overlap area; (4) using minimap2 to compare the corrected reads again; and (5) based on the results of mutual comparison, adopting the string Graph algorithm for assembly. Next, the assembled contigs were polished using Nanopore reads with Racon 1.4.3 [89], followed by secondary polishing with Pilon 1.23 [90] utilizing the Illumina reads. Finally, the redundancy of genomes would be removed after initial assembly and error correction using the software Purge-Haplotigs. Meanwhile, based on the sequence similarity and the proportion of the redundant part in the total length of contigs, the redundant contigs were identified and removed.

#### 4.3.2. Genomic Annotation

In our assembled genome, we carried out genomic annotation, including the prediction of repeated sequences, the prediction of non-coding RNA, and the prediction of gene structure and functional annotation. Repetitive sequence annotation is combined with the homologous prediction method and the de novo ab initio prediction method [91]. The homologous prediction method depended on the RepBase library [92] (http://www.girinst.org/repbase; accessed on 21 March 2020) (software:RepeatMasker [93] and RepeatProteinMask). The de novo ab initio prediction method included its own sequence comparison (RepeatModeler (http://www.repeatmasker.org/RepeatModeler/ accessed on 21 March 2020), Piler [94], RepeatScount (http://bix.ucsd.edu/repeatscout/ accessed on 21 March 2020) and repetitive sequence features (software: TRF (https://tandem.bu.edu/trf/trf.html accessed on 21 March 2020) and LTR-Finder [95]).

The structure prediction of coding genes was usually combined with a variety of prediction methods, such as homolog prediction [96], de novo prediction (software: Augustuss [97], Genscan, GlimmerHMM, etc.), and cDNA/EST prediction, etc. Meanwhile, transcripts were obtained by comparing the RNA-seq data with Tophat and assembled by Cufflinks [72]. Then, a non-redundant and complete gene set was obtained by integrating predicted gene sets based on MAKER software [98] and integrating the CEGMA results based on the HiCESAP process. Finally, based on the foreign protein databases (SwissProt, TrEMBL, KEGG, InterPro and GO), proteins in the gene sets were functionally annotated. Based on tRNA structural characteristics, tRNA sequences in the genome were searched by using the tRNAscan-SE software [99]. Based on the highly conserved characteristics of rRNA, the rRNA sequence was blasted with related species’ rRNA sequences. In addition, miRNA and snRNA sequence information on the genome was predicted using the covariance model of the Rfam family (http://rfam.xfam.org/ accessed on 21 March 2020) [100].

### 4.4. Aniline Blue Staining Assay

The aniline blue staining assay was performed according to the established procedure with minor modifications [54]. The anther was removed the day before flowering. The emasculated stigmas were pollinated with the corresponding pollen on the day of flowering. After 16 h, the pollinated pistils were put into the fixer buffer with anethanol:glacial acetic acid ratio of 3:1 for 2 h, and then the samples were incubated in 1 M NaOH at 65 °C water for 1 h. Next, the pistils were washed three times with distilled water and stained with the basic aniline blue staining (0.1% aniline blue staining in 0.1 M K_3_PO_4_) for 3 h. The stained pistils were placed on the glass slide in distilled water to detect the pollen germination and pollen tube growth using the blue channel (UV 340–380 nm) of a fluorescence microscope (SP8; Leica, Wetzlar, Germany).

### 4.5. RNA Extraction and RT-qPCR

Anthers were cut from medium-size buds (2 to 3 days before flowering) and stigmas were gained from mature buds (on the day of flowering). Total RNA of stigmas and anthers was isolated from more than 30 stigmas and from six anthers of more than 20 buds using the SV Total RNA Isolation System kit (Promega, Madison, WI, USA) following the manufacturer’s instructions. One microgram of total RNA was used for the cDNA synthesis using a PrimeScript^TM^ RT reagent kit (Takara, Tokyo, Japan). The real-time quantitative polymerase chain reaction (RT-qPCR) was performed using 2× SYBR Green master mix (Toyobo, Osaka, Osaka Prefecture, Japan) with the 10 μL reaction system, including 5 μL of 2× SYBR Green master mix, 0.4 μL of each primer at 10 μM and 4.2 μL of 50× diluted cDNA. Amplification was performed using the CFX96 Touch Real-Time PCR Detection System (Bio-Rad, Hercules, CA, USA). The amplification program involved one cycle of 95 °C for 5 min, followed by 45 cycles at 95 °C for 15 s, 60 °C for 20 s, and 72 °C for 30 s. After each run, a melting curve was performed by heating up the samples from 60 to 95 °C. All analyses were repeated with three biological replicates. *Actin* gene (GenBank accession No: AF111812) was used as an internal control and *actin* was used to normalize transcript levels for all expression analyses [101]. Significant differences were calculated by Student’s *t*-test. The primer pairs coming from *S* locus genes (*SRK* and *SCR*) are developed. All primers are listed in Table S15.

### 4.6. Sequence Alignment and Phylogenetic Analysis

CDS and amino acid sequences of class II SRK and SCR were obtained from the NCBI and their GenBank or entry name are listed in Table S13. The promoter sequence of *BrSRK-60* came from the genome of ‘Chiifu’ [32]. MEGA-X [102] program was used to perform multiple sequence alignment, and the results were modified with geneDoc (http://nrbsc.org/gfx/genedoc 26 August 2015). Class II SCR drafts were drawn referring to the research results [75] and class II SRK drafts were drawn referring to the report [74]. Phylogenetic trees were constructed using MEGA-X software [102] using the neighbor-joining method and a bootstrap test that was replicated 1000 times. 

### 4.7. Synteny Assay of Two Class II S Locus

To display the synteny relationships of two class II *S* haplotypes, *BrS-60* was obtained from the genome of ‘Chiifu’ [32] and ‘Z1’ [33], which possessed the same *S* haplotype and a contrasting SI phenotype, and *BrS-325* was obtained from our assembled genome. Collinearity analysis of *S* locus sequences was performed using the software: MuMmer [103] and JCVI [104]. The *S* locus collinearity analysis of *BrS-325* and *BrS-60* was performed with the MuMmer tool. Then, visualizations of the *S* locus collinearity analysis were displayed using a JCVI tool.

### 4.8. Self-Incompatibility Phenotype Assay

The self-incompatibility phenotype was measured as in [105]. When three to five flowers were set on the major inflorescence, a bag was used to cover the major inflorescence and two or three secondary branches after cutting the opened flower and the apical buds for self-pollination. The bags were administered gently every two days to facilitate self-pollination. After two weeks, the bags were removed to allow the growth of seeds. After seeds were mature, the number of seeds was counted, and the self-compatibility index (SCI) was calculated as the ratio of the number of seeds to the number of flowers [106]. Plants with SCI ≥ 2 were referred to as self-compatible, and plants with SCI < 2 were considered self-incompatible. The significant difference was calculated by Student’s *t*-test.

## 5. Conclusions

In conclusion, we identified a new class II *S* haplotype in *Brassica*, which displayed completely self-compatibility. It is a good resource for breeding practices due to its recessiveness to all class I *S* haplotypes [20]. Here, we gained a high-quality reference genome, which provides the opportunity to carry out comparative genomics research on different traits [66]. In this study, compared with other class II SRK and SCR, we found many amino acid variations in BrSRK-325 and BrSCR-325, which may influence the interaction between the ligand and the receptor. Based on the above results, we inference that amino acid variations in critical sites may lead to self-incompatibility destruction in a new class II *S* haplotype in *B. rapa*.

## Figures and Tables

**Table 1:** Genetic analysis of the SC traits in line ‘325’.

Material	Genotype			SI/SC	Expected	χ^2^
	I	I/II	II	Phenotype	Ratio	
325	0	0	12	0/12		
326	12	0	0	12/0		
325 × 326	0	12	0	12/0		
326 × 325	0	12	0	12/0		
(325 × 326)F_2_	20	50	22	70/22	3:1	0.094 < 3.84

**Table 2:** Comparisons between the assembly and annotations of the pak choi ‘325’ and published pak choi ‘ZYCX’, Chinese cabbage ‘Chiifu’ and yellow sarson ‘Z1’ genome assemblies.

	325	ZYCX	Chiifu	Z1
Assembly feature				
Total assembly size (Mb)	376.69	370.42	353.14	401.92
Contig number	314	1985	1498	1037
Contig N50 (Mb)	4.54	2.82	1.45	2.27
Longest length (Mb)	22.32	22.37	9.42	22.13
Genome annotation				
Gene model	42500	45363	46250	46721
Percentage of anchored genes (%)	0.9691	0.985	0.9858	0.9814

**Table 3:** Comparative analysis of the *S* locus between two class II *S* haplotype, *BrS-60* and *BrS-325.*

Accession	Phenotype	*S* haplotype	Alignment Size (kb)	*S* locus Size (kb)	Distance 1 ^a^ (kb)	Distance 2 ^b^ (kb)	Distance 3 ^c^ (kb)	SCR Length (bp)	SRK Length (bp)		SLG Length (bp)	TE Type
325	SC	*BrS-325*	145.991	67.777	41.173	19.706	15.384	391	5618 ^d^	6083 ^e^	4122	DNA-TE
Z1	SC	*BrS-60*	147.921	54.448	24.828	6.575	11.533	378	-	6720	1966	LTR-TE
Chiifu	SI	*BrS-60*	155.427	73.764	41.492	6.582	26.943	378	-	7967	1459	LTR-TE

a. Intergenic distance between *SCR* and *SLG*. b. Intergenic distance between *SCR* and *SRK*. c. Intergenic distance between *SRK* and *SLG*. *BrSRK-325b* was used as the reference gene in *BrS-325* locus to calculate the intergenic distance. d. Gene length of *BrSRK-325a*. e. Gene length of *BrSRK-325b*.

## Data Availability

All raw sequencing data of the genome are available at NCBI under accession number PRJNA782472.

## Supplementary Materials

The following are available online at https://www.mdpi.com/article/10.3390/plants10122815/s1: Figure S1: Multiple nucleotide sequence alignment of the class II *SCR*, Figure S2: Multiple nucleotide sequence alignment of the first exon in class II *SRKs*, Figure S3: Promoter sequence alignment of class II *SRKs*, Figure S4: Multiple amino acid sequence alignment of class II SRKs, Table S1: Statistics for the genome assembly results of the self-compatible line ‘325’, Table S2: Statistics for the sequencing results of the genome assembly, Table S3: Blasting of the second-generation reads and third-generation reads of the genome assembly, Table S4: Statistics of genome completeness in the SC pak choi genome assemblies according to BUSCO, Table S5: Statistics of repetitive sequences in the self-compatible line ‘325’, Table S6: Statistics of gene annotation in the self-compatible line ‘325’, Table S7: Statistics the genes functional annotation in the self-compatible line ‘325’, Table S8: Statistics of non-coding RNA annotation in the self-compatible line ‘325’, Table S9: Statistics of gene annotation completeness in the SC pak choi assembled genome according to BUSCO, Table S10: Function prediction of *S* locus and boundary regions in *BrS-325*, Table S11: Amino acid sequence similarity of class II SCR, Table S12: Amino acid sequence similarity of class II SRK. Table S13: The gene information of the known class II SRK and SCR in *B. rapa* and *B. oleracea*, Table S14: Primers designed to detect the *S* haplotypes based on the *SLG*, *SRK* and *SCR* sequence, Table S15: Primers designed to detect the expression of *SRK* and *SCR.*
