# Supplementary material for: Genetic and Molecular Characterization of a Self-Compatible Brassica rapa Line Possessing a New Class II S Haplotype
Source: Plants (Basel). 2021 Dec 20;10(12):2815. doi: 10.3390/plants10122815 (PMC8709392; doi:10.3390/plants10122815)
Supplement: Supplementary file 1 [file plants-10-02815-s001.zip › plants-1471693-supplementary.pdf]

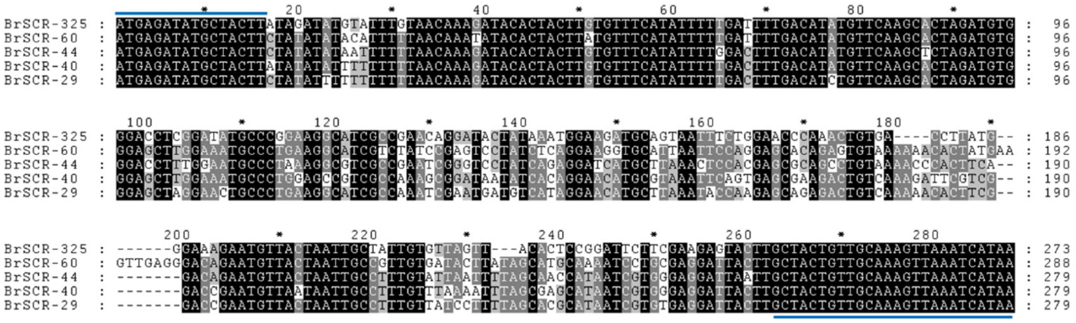

Figure S1. Multiple nucleotide sequence alignment of the class II *SCRs.*

The blue overline and underline indicate the specific primer pairs for cloning class II *SCRs.*

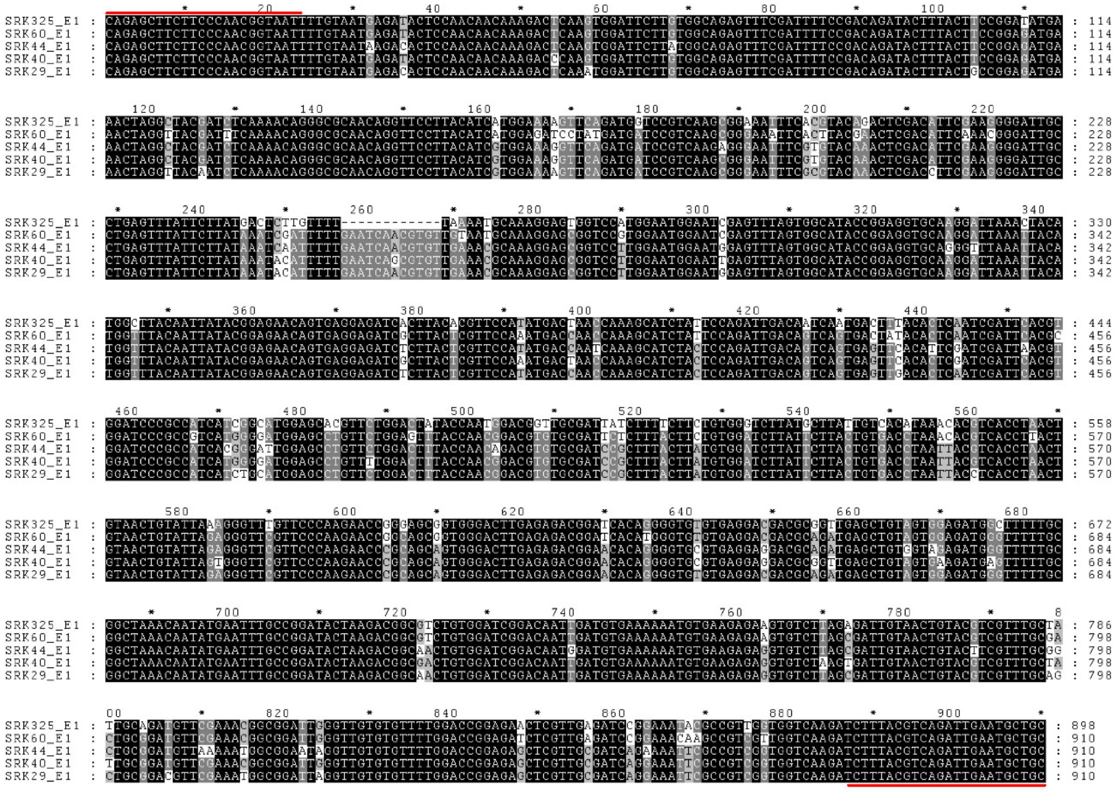

Figure S2. Multiple nucleotide sequence alignment of the first exon in class II *SRKs.*

The red overline and underline indicate the specific primer pairs for cloning the first exon in class II *SRKs*.

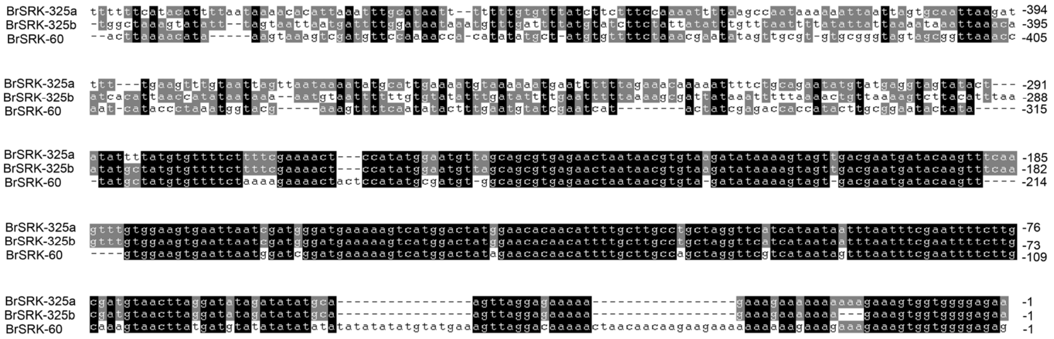

Figure S3. Promoter sequencealignment of class II *SRKs.*

500 bp promoter sequence alignment of *BrSRK-325a/b* and *BrSRK-60*. The black padding indicated conserved nucleotides.

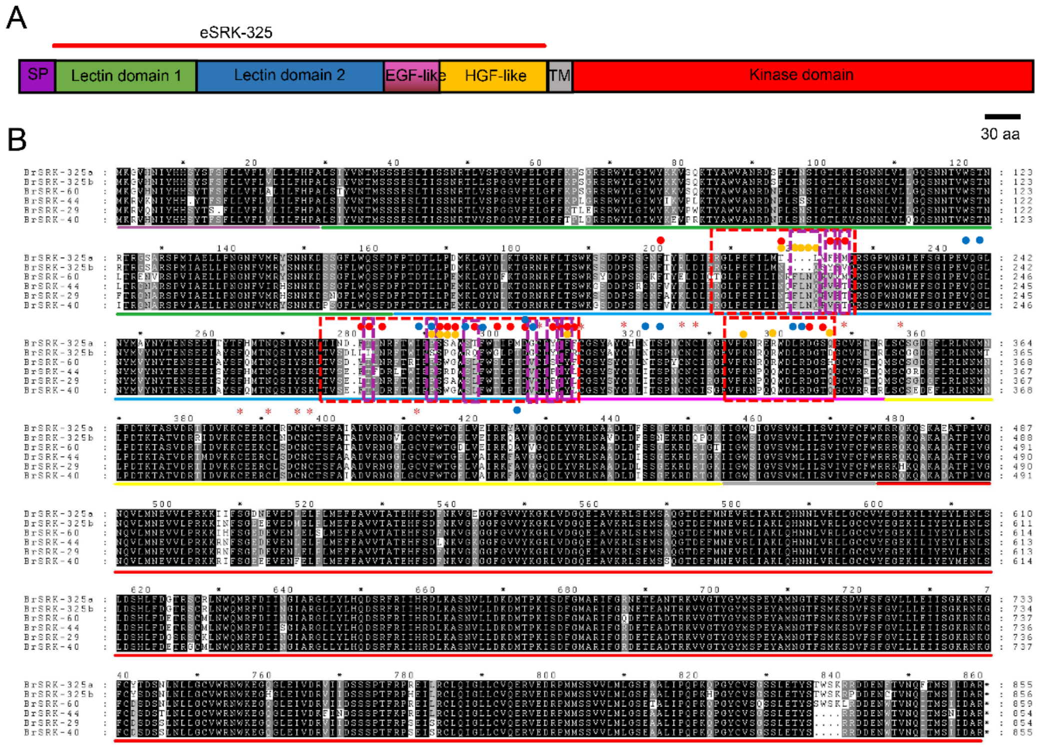

Figure S4. Multiple amino acid sequence alignment of class II SRKs.(**A**)The structure drawing of BrSRK-325a. The BreSRK-325a was comprised of Lectin domain 1, Lectin domain 2, EGF-like and HGF-like. (**B**) Sequence alignment of class II SRKs. The different color underlines represented the aforementioned domains in (**A**). The conserved residues were highligthed in black padding. The hypervariable regions (HV I, HV II, HV III) sequence alignment of class II SRK. Above the alignment, contact amino acids against the cognate SCR were shown red circles, based on the same sites contacting amino acids of BrSRK-8 and BrSRK-9 and yellow circles, based on the contacting amino acids of class II SRK. The blue circles indicated the residues involved in SRK homo-dimerization. The variable amino acids of BrSRK-325comparing with other class II SRK were shown by purple dotted boxes.

Table S1. Statistics for the genome assembly results of the self-compatible line '325'.

Mode	Total length (bp)	Total number	Total number (>= 2 kb)	max length(bp)	N50 (bp)	N90 (bp)	GC content(%)
NextDenovo assembly[a]	440,847,141	547	547	22,190,325	2,715,399	283,704	37.3
assembly+racon[b]	442,737,300	547	547	22,291,483	2,729,615	285,420	37.3
assembly+racon+pilon[c]	443,142,766	547	547	22,322,820	2,732,951	285,731	37.3

purge_haplotigs[d]	376,699,982	314	314	22,322,776	4,545,633	414,265	36.8

a：The three-generation reads were assembled by NextDenovo. b: The third-generation polish was corrected by racon. c：The second-generation polish was corrected by pilon. d: The corrected assembly genome was removed redundancy by purge-haplotigs.

Table S2. Statistic for the sequencing results of the genome assembly.

	Contig Length(bp)	Contig Number	Scaffold Length(bp)	Scaffold Number
N90	414,265	153	414,265	153
N80	808,654	85	808,654	85
N70	1,415,163	49	1,415,163	49
N60	2,670,592	29	2,670,592	29
N50	4,545,633	19	4,545,633	19
Total length	376,699,982	-	376,699,982	-
Number(>=100bp)	-	314	-	314
Number(>=2kb)	-	314	-	314
Max length	22,322,776	-	22,322,776	

Table S3. Blasting the second-generation reads and third-generation reads of the genome assembly.

Comparison reads	Sample name	Mapping rate(%)	Average sequencing depth	Coverage (%)	Coverage at least 4X(%)	Coverage at least 10X(%)	Coverage at least 20X(%)
Illumina	325	99.39	175	97.79	96.97	96.18	95.32
Nanopore	325	99.99	192.32	99.98	99.89	99.75	99.54

Table S4. Statistics of genome completeness in the SC pak choi genome assemblies according to BUSCO.

Type	Proteins	Percentage(%)
Complete BUSCOs (C)	1,404	97.5
Complete and single-copy BUSCOs (S)	1,219	84.65
Complete and duplicated BUSCOs (D)	185	12.85
Fragmented BUSCOs (F)	11	0.76
Missing BUSCOs (M)	25	1.74
Total BUSCO groups searched	1,440	100

Table S5. Statistics of repetitive sequences in the self-compatible line '325'.

	RepBase TEs		TE Proteins		De novo		Combined TEs	
Type	Length (bp)	%in Genome	Length (bp)	%in Genome	Length (bp)	%in Genome	Length (bp)	%in Genome
DNA	15,508,528	4.12	4,125,037	1.1	51,954,234	13.79	58,747,051	15.6
LINE	14,419,054	3.83	7,504,021	1.99	13,888,988	3.69	24,355,860	6.47
SINE	28,364	0.01	0	0	221,319	0.06	249,650	0.07
LTR	43,712,246	11.6	27,079,092	7.19	115,232,035	30.59	120,387,356	31.96
Satellite	13,328,924	3.54	0	0	2,782,621	0.74	14,747,078	3.91
Simple_repeat	0	0	0	0	270	0	270	0
Other	1,053	0	231	0	0	0	1,284	0
Unknown	74,781	0.02	16,668	0	12,768,814	3.39	12,850,035	3.41
Total	86,487,370	22.96	38,719,738	10.28	184,646,876	49.02	198,340,146	52.65

Table S6. Statistics of gene annotation in the self-compatible line '325'.

Gene set	Protein coding gene number	Average gene length (bp)	Average CDS length (bp)	Average exon per gene	Average exon length (bp)	Average intron length (bp)
denovo/GlimmmerHMM	60,336	2,513	1,717	3.66	468.9	299.17
denovo/AUGUSTUS	46,407	1,959	1,122	5.18	216.73	200.44
homo/Brassica_rapa	36,781	2,560	962.24	3.46	278.34	650.41
homo/Arabidopsis_thaliana	57,090	1,945	802.91	3.16	253.73	527.6
homo/Brassica_oleracea_var_oleracea	28,367	2,681	934.64	3.57	262.15	680.76
homo/Arabidopsis_lyrata_subsp_lyrata	47,154	2,439	907.79	3.3	274.71	664.47
homo/Brassica_napus	30,295	5,817	913.98	2.95	309.44	2,510
trans.orf/RNAseq	22,374	2,841	1,254	6.28	291.93	190.49
BUSCO	1,619	2,676	1,546	8	193.32	161.38
MAKER	38,308	2,912	1,169	5.29	371.92	220.7
HiCESAP	43,855	2,650	1,121	5	361.02	211.69

Table S7. Statistics the genes functional annotation in the self-compatible line '325'.

			Number	Percent (%)
Total			43,855	
	Annotated		42,500	96.91
		InterPro	32,788	74.76
		GO	22,496	51.3
		KEGG_ALL	41,373	94.34
		KEGG_KO	15,174	34.6
		Swissprot	28,849	65.78
		TrEMBL	42,255	96.35
		TF	3,067	6.99
		Pfam	32,022	73.02
		NR	42,132	96.07
		KOG	35,718	81.45
	Unannotated		1,355	3.09

Table S8. Statistics of non-coding RNA annotation in the self-compatible line '325'.

Type		Copy	Average length(bp)	Total length(bp)	% of genome
miRNA		241	129	31,143	0.008267
tRNA		1,431	75	107,296	0.028483
rRNA	rRNA	1,623	466	756,185	0.200739
	18S	514	1,136	584,105	0.155058
	28S	381	194	73,932	0.019626
	5.8S	328	155	50,704	0.01346
	5S	400	119	47,444	0.012595
snRNA	snRNA	1,397	112	156,197	0.041465
	CD-box	1,118	106	118,073	0.031344
	HACA-box	102	127	12,961	0.003441
	splicing	174	143	24,847	0.006596
	scaRNA	3	105	316	0.000084

Table S9. Statistics of gene annotation completeness in the SC pak choi assembled genome according to BUSCO.

Type	Proteins	Percentage (%)

Complete BUSCOs	1,411	98
Complete Single-Copy BUSCOs	1,205	83.7
Complete Duplicated BUSCOs	206	14.3
Fragmented BUSCOs	12	0.8
Missing BUSCOs	17	1.2
Total BUSCO groups searched	1,440	100

Table S10. Functions prediction of *S* locus and boundary regions in *BrS-325.*

Gene ID	Prediction function
Bra0024300.1	Putative transcription factor bHLH086 (AtbHLH86)
Bra0024310.1	Domain of unknown function (DUF4228)
Bra0024320.1	Polyadenylation and cleavage factor homolog 5
Bra0024330.1	A1 cistron-splicing factor AAR2
Bra0024340.1	Methionyl-tRNA formyltransferase
Bra0024350.1	A S-adenosyl-L-methionine:carboxyl methyltransferase
Bra0024360.1	E3 ubiquitin-protein ligase SINA-like 4
Bra0024370.1	ATP-dependent Clp protease proteolytic subunit 3
Bra0024380.1	Protein-lysine N-methyltransferase
Bra0024385.1	S locus protein 11
Bra0024390.1	*S* receptor kinase
Bra0024395.1	*S* receptor kinase
Bra0024396.1	*S*-locus-specific glycoprotein
Bra0024400.1	SAM dependent carboxyl methyltransferase
Bra0024410.1	DNA ligase 6 Brassica rapa
Bra0024420.1	Anti-silencing function 1a protein (S-locus protein 7)
Bra0024430.1	Cyclin-dependent kinase D-2(CDK2)
Bra0024440.1	Protein DETOXIFICATION 7 (MATE protein 7)
Bra0024450.1	Protein DETOXIFICATION 7 (MATE protein 7)
Bra0024460.1	Protein DETOXIFICATION 9 (MATE protein 9)

Table S11. Amino acid sequence similarity of class II SCR.

Name	BrSCR-325	BrSCR-60	BrSCR-44	BrSCR-40	BrSCR-29
BrSCR-325	100%				
BrSCR-60	59.38%	100%			
BrSCR-44	56.99%	64.58%	100%		
BrSCR-40	58.06%	63.54%	68.82%	100%	
BrSCR-29	59.14%	64.58%	69.89%	73.12%	100%

Table S12. Amino acid sequence similarity of class II SRK.

Name	BrSRK-325a	BrSRK-325b	BrSRK-60	BrSRK-44	BrSRK-40	BrSRK-29
BrSRK-325a	100%					
BrSRK-325b	92.88%	100%				
BrSRK-60	90.23%	89.90%	100%			
BrSRK-44	87.67%	87.92%	90.58%	100%		
BrSRK-40	88.95%	89.08%	91.40%	94.28%	100%	
BrSRK-29	89.19%	89.31%	91.51%	94.62%	91.40%	100%

Table S13. The genes information of the known class II SRK and SCR in *B. rapa* and *B. oleracea.*

gene name	GenBank/entry name	gene name	GenBank/entry name
BrSCR-29	AB067449.1	*BrSRK-29*	AB008191.1
BrSCR-40	AB067450.1	*BrSRK-40*	AB211197.1
BrSCR-44	AB067451.1	*BrSRK-44*	AB211198.1
BrSCR-60	AB067446.1	*BrSRK-60*	AB032474.1
BoSCR-15	AB024416	*BoSRK15*	Q9XFW5/Y18259
BoSCR-5	Q8S9C1	*BoSRK-5*	Y18259/Q9T0M1
BoSCR-2b	Q8S9C2	*BoSRK-2b*	AB024416

Table S14. Primers designed to detect the *S* haplotypes based on the *SLG*, *SRK* and *SCR* sequence.

No	primer	sequence(5'-3')	length	source	S haplotype	Species
1	PS5	ATGAAAGGCGTAAGAAAAACCTA	23	*SLG-8* (1–23)	class I	*B. rapa*
	PS15	CCGTGTTTTATTTTAAGAGAAAGAGCT	27	*SLG-6* (1,336–1,310)	class I	*B. oleracea*
2	PK1	CTGCTGATCATGTTCTGCCTCTGG	24	Second exon of *SRK* gene	class I	*B. oleracea*
	PK4	CAATCCCAAAATCCGAGATCT	21	Fifth exon of *SRK* gene	class I	*B. oleracea*
3	PS3	ATGAAAGGGGTACAGAACAT	20	*SLG-2A* (1–20)	class II	*B. oleracea*
	PS21	CTCAAGTCCCACTGCTGCGG	20	*SLG-2A* (1,025–1,006)	class II	*B. oleracea*
4	SRKII-E1L	CAGAGCTTCTTCCCAACGGTAATT	24	*SRK-60* (404-427)	class II	*B. rapa*
	SRKII-E1R	GCAGCATTCAATCTGACGTAAAGA	24	*SRK-60* (1290-1313)	class II	*B. rapa*
5	SRKII-E4L	TCTTGTCCGACTTCTTGGCTGTT	23	*SRK-60* (6894-6916)	class II	*B. rapa*
	SRKII-E7R	CGTCAATGATCGACATGGTGATT	23	*SRK-60* (7928-7950)	class II	*B. rapa*
6	IISCR-1L	TCATAAGTCATGAGATATGCTACTT	25	*SCR-60* (-9-16)	class II	*B. rapa*
	IISCR-1R	TTATGATTTAACTTTGCAACAGTAGC	26	*SCR-60* (260-288)	class II	*B. rapa*

Table S15. Primers designed to detect the expression of *SRK* and *SCR*.

No	primer	sequence(5'-3')	length	Use
1	SRK-12 RT-1F	AGGATATCCGGAATTACATTGGCAAC	26	*BrSRK-12*
	SRK-12 RT-1R	GATTGTGTCATCCCGTTCATAAGTAC	26	
2	SCR-12 RT-1F	AGTCGTTCTCAAGAATTAACAGAAG	25	*BrSCR-12*
	SCR-12 RT-1R	TTAGCATTTACATGTACAAAGCCGTC	26	
3	SCR-325 RT-1F	TGTAACAAAGATACACTACTTGTGT	25	*BrSCR-325*
	SCR-325 RT-1R	ACTCTTCGAAGAATCCGGAGTGT	23	
4	SRK-325b RT-1F	CGGCTGATGTTCGAAATGGAGT	22	*BrSRK-325b*
	SRK-325b RT-1R	CCAGGTTGGTCCCTCTTCTCATTC	21	
5	SRK-325a RT-1F	GCAGATGTTCGAAACGGCGG	20	*BrSRK-325a*
	SRK-325a RT-1R	CAGTTCGGTCTCTCTTCTCACC	22	
6	SRK-325 RT-1F	TGGTCAAGATCTTTACGTCAGATTG	25	*BrSRK-325*
	SRK-325 RT-1R	GCTTGCTTCTGTCTCCTCTTCC	22	
7	Actin-1F	CTATCCTCCGTCTCGATCTCGC	22	*Actin 7*
	Actin-1R	CTTAGCCGTCTCCAGCTCTTGC	22	
